# MPT64 assays for the rapid detection of *Mycobacterium tuberculosis*

**DOI:** 10.1186/s12879-021-06022-w

**Published:** 2021-04-10

**Authors:** Xun-Jie Cao, Ya-Ping Li, Jia-Ying Wang, Jie Zhou, Xu-Guang Guo

**Affiliations:** 1 Department of Clinical Laboratory Medicine, The Third Affiliated Hospital of Guangzhou Medical University, Guangzhou, 510150 China; 2 Department of Clinical Medicine, The Third Clinical School of Guangzhou Medical University, Guangzhou, 511436 China; 3 Department of Clinical Medicine, The Second Clinical School of Guangzhou Medical University, Guangzhou, 511436 China; 4 Key Laboratory for Major Obstetric Diseases of Guangdong Province, The Third Affiliated Hospital of Guangzhou Medical University, Guangzhou, 510150 China; 5 Key Laboratory of Reproduction and Genetics of Guangdong Higher Education Institutes, The Third Affiliated Hospital of Guangzhou Medical University, Guangzhou, 510150 China

**Keywords:** MPT64, *Mycobacterium tuberculosis*, Tuberculosis, MTB, Commercial kits

## Abstract

**Background:**

Tuberculosis (TB) is a serious infectious disease caused by *Mycobacterium tuberculosis* (MTB). An estimated 1.7 billion people worldwide are infected with *Mycobacterium tuberculosis* (LTBI) during the incubation period without any obvious symptoms. Because of MTB’s high infection and mortality rates, there is an urgent need to develop a fast, portable, and sensitive diagnostic technology for its detection.

**Methods:**

We included research from PubMed, Cochrane Library, Web of Science, and Embase and extracted the data. MetaDisc and STATA were used to build forest plots, Deek’s funnel plot, Fagan plot, and bivariate boxplot for analysis.

**Results:**

Forty-six articles were analyzed, the results of which are as follows: sensitivity and specificity were 0.92 (0.91–0.93) and 0.95 (0.94–0.95) respectively. The NLR and PLR were 0.04 (95% CI 0.03–0.07) and 25.32 (95% CI 12.38–51.78) respectively. DOR was 639.60 (243.04–1683.18). The area under the SROC curve (AUC) was 0.99.

**Conclusions:**

MPT64 exhibits good diagnostic efficiency for MTB. There is no obvious heterogeneity between the three commercial kits.

**Supplementary Information:**

The online version contains supplementary material available at 10.1186/s12879-021-06022-w.

## Introduction

Tuberculosis (TB) is a serious infectious disease caused by *Mycobacterium tuberculosis* (MTB). The Global Tuberculosis Report 2019 stated that in 2018, about 1.5 million people worldwide died of TB and nearly 10 million people died from MTB, of which only 6.4 million were diagnosed and officially reported. An estimated 1.7 billion people worldwide are infected with MTB (LTBI) during the incubation period without any obvious symptoms [1]. TB mainly damages the lungs, causing lung disease or pulmonary tuberculosis, but it can also damage other organs, causing bone tuberculosis, nerve tuberculosis, skin tuberculosis, kidney tuberculosis, and other infections [2].

The incubation period of TB is related to the immune status of the person, and there is no clinical, radiological, or microbiological evidence of active TB disease during the incubation period [3]. The typical symptoms of active TB are chronic cough, bloody sputum, night sweats, fever, and weight loss and various symptoms can be observed in extrapulmonary cases [4]. The conventional technique for detecting MTB in an analytical sample (such as pus, sputum, or tissue biopsy) takes two to 6 weeks. So far, for the rapid detection of MTB, many techniques have been developed, such as ELISA (enzyme-linked immunosorbent assay), real-time polymerase chain reaction (PCR), latex agglutination, Gen-Probe amplified M. Tuberculosis direct test, and flow cytometry [5]. Compared to traditional microbial culture techniques, these methods exhibit higher sensitivity in a shorter time, but this requires advanced laboratories and technicians, which is the main limitation of these methods. Therefore, it is essential to develop a real-time, portable, and sensitive technology that can quickly detect MTB at an affordable cost.

MPT64, which is a 24-kDa protein of MTB and an important secretory protein of pathogenic bacteria, is often used as a candidate protein for diagnosis and in vaccines [6, 7]. At present, there are many ways to detect the MPT64 protein, such as immunochromatography (ICT), ELISA, SD Bioline, and Capilia TB [8–11].

To date, many studies have evaluated the diagnostic accuracy of MPT64 for MTB. In 2013, a systematic review evaluated the diagnostic accuracy of commercial MPT64-based tests for MTB [12]. Our purpose was to evaluate the efficacy of MPT64 protein as a target for detection of *Mycobacterium tuberculosis* infection. What’s more, we also evaluated the diagnostic efficacy of three common commercial kits relying on MPT64 antigen assay. Our study was more comprehensively than the study by Yin et al [12]

## Methods

### Research identification and selection

Three independent reviewers (XJ Cao, YP Li, JY Wang) searched four online electronic databases up to July 15, 2020. The databases searched included Embase, Cochrane Library, PubMed, and Web of Science. Finally, we retrieved 1222 articles. After deleting the repetitive articles, 521 were left; 64 studies were left after eliminating unrelated studies and reviews. We included articles that met the expected requirements: (1) The data was provided as two-by-two tables and (2) full text publications and (3) used at least one accepted reference standard (biochemical method or molecular methods). The exclusion criteria consisted of the following: (1) studies whose samples were less than 10 to avoid selection bias, (2) meta-analyses, meeting summaries, and systematic reviews, and (3) animal research. There were 49 studies that successfully extracted the two-by-two tables.

### Quality assessment and data extraction

For each eligible article, two investigators (XJ Cao and YP Li) independently extracted the following information: the first author, year of publication, MPT64 detection method, reference standard used, methodological quality, and data for the two-by two tables. Any disagreements were resolved via discussion with the third investigator (JY Wang).

According to the Quality Assessment of Diagnostic Accuracy Studies tool-2 (QUADAS-2), recommended by the Cochrane Collaboration, two investigators independently reviewed the methodological quality of the eligible articles [13]. Disagreements were resolved by consensus. Revman 5.3 was used to perform the quality assessment.

### Statistical analysis

In order to analyze the summary estimation of MPT64, we constructed the MPT64 test to cross-classify the two-by-two tables. True Positive (TP), True Negative (TN), False Positive (FP), and False Negative (FN) were directly extracted from the original research or obtained by calculation. The forest plots were used to evaluate the sensitivity and specificity of each study, with a 95% confidence interval (95% CIs). The summary receiver operating characteristic (SROC) curve was established to summarize the combined distribution of sensitivity and specificity. The area under the SROC curve (AUC) was used to evaluate the accuracy of the overall test. Moreover, the combined SPE and SEN were also used to calculate the negative likelihood ratio (NLR) and positive likelihood ratio (PLR). The calculation method of NLR is false negative rate (1 sensitivity) divided by true negative rate (specificity). When a test finding is negative, the NLR is used to determine the degree of decreasing false-negative risk for the test, and evaluate the commercial kits diagnostic accuracy [14]. The diagnostic odds ratio (DOR) was also used for analysis which was an easily comparable measure to get the tool validity. DOR not only combines the advantages of SPE and SEN, but also has superior accuracy as a single indicator [15]. The Fagan plot was constructed to show the relationship between the pre-probability, likelihood ratio, and post-probability. The Deek’s funnel plot was constructed to visually check any potential publication bias. The Fagan plot was constructed to show the relationship between the former probability, likelihood ratio, and latter probability. Moreover, in order to perform heterogeneity testing, a bivariate boxplot was constructed.

To explore the reasons for the heterogeneity and the accuracy of the detection of the three kits, we conducted a subgroup analysis of the studies in which the detection method was SD Bioline, Capilia TB, or BD MGIT TBcID. First, we divided the research that used the three kits into one subgroup and those that used other detection methods into another subgroup. Then, we divided “the three-kits group” into three groups: SD Bioline, Capilia TB, and BD MGIT TBcID. Furthermore, the bivariate boxplot was also drawn to assess the overall heterogeneity. Publication bias was tested using the funnel plot.

The analyses were performed using the Stata statistical software package, version 12.0 (Stata Corp LP, College Station, U.S.A.), Review Manager 5.3, and Meta-DiSc 1.4.

## Results

### Inclusion and exclusion criteria and quality assessment

**Fig. 1:**
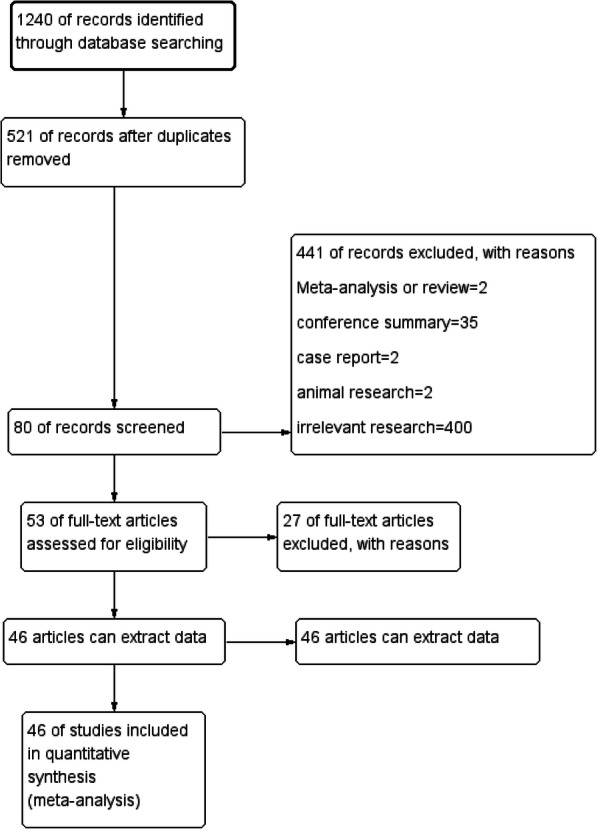
Flow diagram of study identification and inclusion

**Fig. 2:**
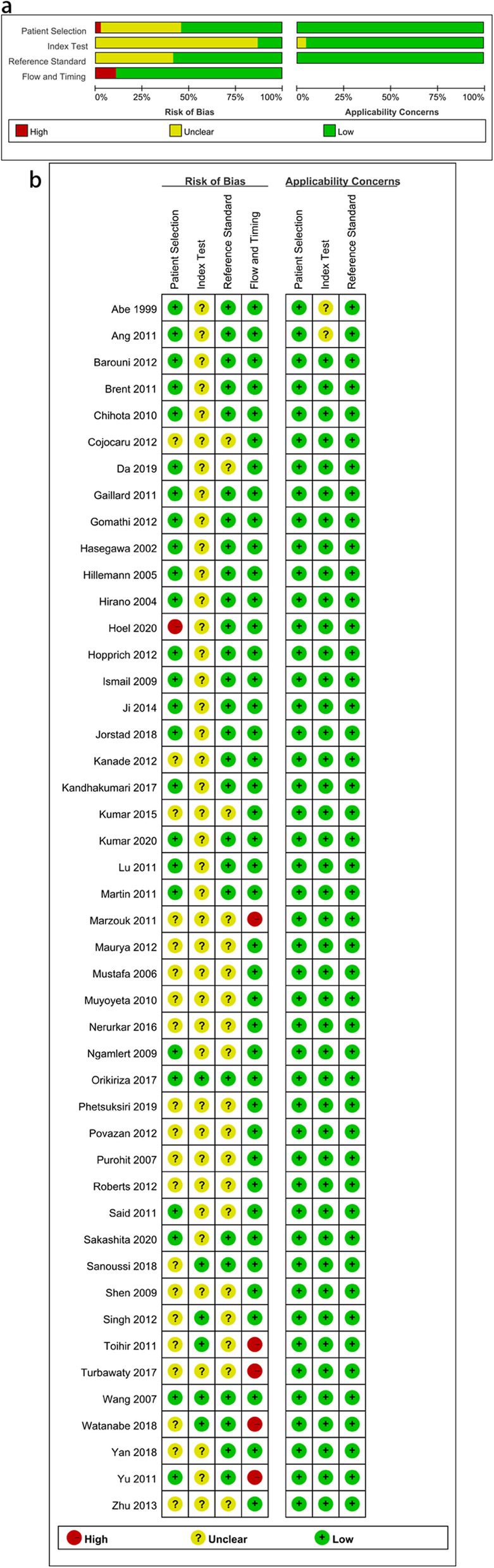
Quality assessment of the included studies. **a**. Overall quality assessment of the included studies, **b**. Quality assessment of the individual studies

We searched a total of 1240 records identified through the database searches. After removing duplicate records, we obtained 521 records. Then 441 were excluded; these consisted of two meta-analyses or reviews, thirty-five conference summaries, two case reports, two animal-based research, and four hundred irrelevant studies. We screened 80 records. After excluding 27 full-text articles for reasons, we assessed 53 good-quality full-text articles for eligibility. Finally, data was extracted from 46 articles analysis. The flow diagram is shown in Fig. 1. The characteristics of the studies included in the articles are shown in Table 1. The quality assessment of the included studies is shown in Fig. 2.

**Table 1:** Characteristics of the studies included in the articles

Author	Study	Study Design	Reference Test	Sample size	Medium	Method of detection
Hoel, I	Hoel 2020 [16]	Cross Sectional Study	composite reference standard (CRS)	288	liquid	ICC Staining (Dako Envision + System-HRP kit)
Kumar, C	Kumar2020 [17]	Cross Sectional Study	Duplex PCR assay	92	liquid	BD MGIT TBcID
Sakashita, K	Sakashita2020 [9]	Cross Sectional Study	bacteriologically diagnosed	80	solid	ELISA
Da, S	Da 2019 [18]	Cross Sectional Study	CRS	68	liquid	ELISA
Phetsuksiri, B	Phetsuksiri 2019 [10]	Cross Sectional Study	Culture followed by identification of MTC	151	liquid	SD Bioline
Yan, Z	Yan 2018 [19]	Cross Sectional Study	CRS	352	unclear	BD OptEIAe Reagent Set B ELISA kit
Sanoussi, C	Sanoussi2018 [20]	Cross Sectional Study	spoligotyping or PNB/catalase	327	solid	SD Bioline
Jorstad, M	Jorstad 2018 [21]	Cross Sectional Study	CRS	126	Löwenstein–Jensen medium	t 1/250 dilution and Dako kit
Watanabe, P	Watanabe 2018 [22]	Cross Sectional Study	phenotypic techniques and molecular tests(such as conventional or real-time PCR, line probe assays and in-house (PCR and restriction-enzyme analysis) PRA-hsp65 molecular assay)	375	liquid/solid	SD Bioline
Turbawaty, D	Turbawaty 2017 [23]	Cross Sectional Study	acid-fast bacilli and mycobacterial culture	141	liquid	ICT
Kandhakumari, G	Kandhakumari 2017 [24]	Cross Sectional Study	Biochemistry method	75	solid	BD MGIT TBcID
Kandhakumari, G	Kandhakumari 2017 [24]	Cross Sectional Study	Biochemistry method	75	solid	SD Bioline
Orikiriza, P	Orikiriza 2017 [25]	Cross Sectional Study	Biochemistry method/Culturing of mycobacteria	188	liquid	SD Bioline
Nerurkar, V	Nerurkar 2016 [26]	Cross Sectional Study	Culturing of mycobacteria	1093	liquid	SD Bioline
Kumar, N	Kumar 2015 [8]	Cross Sectional Study	Biochemistry method/Molecular method(PNB inhibition test)	484	Solid/liquid	SD Bioline/BD MGIT/Capilia TB
Ji, M	Ji 2014 [27]	Cross Sectional Study	Culturing of mycobacteria	504	liquid	ELISA
Zhu, C^a^	Zhu 2013 [28]	Cross Sectional Study	Biochemistry method/Culturing	328	solid	ELISA
Zhu, C^a^	Zhu 2013 [28]	Cross Sectional Study	Biochemistry method/Culturing	160	solid	ELISA
Hopprich, R	Hopprich 2012 [29]	Cross Sectional Study	Molecular method +Biochemistry method	200	liquid	SD Bioline
Kanade, S	Kanade 2012 [30]	Cross Sectional Study	molecular method	150	solid	SD Bioline
Roberts, S	Roberts 2012 [31]	Cross Sectional Study	molecular method	83	liquid	BD MGIT TBcID
Singh, A	Singh 2012 [32]	Cross Sectional Study	Culturing	161	liquid	SD Bioline
Martin, A	Martin 2011 [33]	Cross Sectional Study	molecular method	131	liquid	BD MGIT TBcID
Marzouk, M	Marzouk 2011 [34]	Cross Sectional Study	Biochemistry method/Culturing	238	Solid/liquid	SD Bioline
Ang, C	Ang 2011 [35]	Cross Sectional Study	Biochemistry method/Culturing	294	Solid/liquid	SD Bioline
Yu, M	Yu 2011 [36]	Cross Sectional Study	Biochemistry method/Culturing	210	liquid	BD MGIT TBcID
Purohit, M	Purohit 2007 [37]	Cross Sectional Study	molecular method	203	solid	DakoCytomation
Mustafa, T	Mustafa 2006 [38]	Cross Sectional Study	molecular method	55	liquid	NA
Hirano, K	Hirano 2004 [39]	Cross Sectional Study	molecular method	545	liquid	Capilia TB
Hasegawa, N.	Hasegawa 2002 [40]	Cross Sectional Study	molecular method or Biochemistry method	304	liquid	BD MGIT TBcID
Abe, C	Abe 1999 [41]	Cross Sectional Study	molecular method	108	liquid	NA
Gomathi, N	Gomathi 2012 [11]	Cross Sectional Study	Biochemistry method	346	Liquid	Capilia TB
Maurya, A	Maurya 2012 [42]	Cross Sectional Study	Biochemistry method	150	Liquid	SD Bioline
Povazan, A	Povazan 2012 [43]	Cross Sectional Study	Biochemistry method	123	Liquid	BD MGIT TBcID
Barouni, A S	Barouni, A S 2012 [44]	Cross Sectional Study	Biochemistry method	161	Liquid	BD MGIT TBcID
Cojocaru, Elena	Cojocaru 2012 [45]	Cross Sectional Study	Biochemistry method	47	Liquid/Solid	SD Bioline
Brent, A	Brent 2011 [46]	Cross Sectional Study	molecular method	208	liquid	BD MGIT TBcID
Gaillard, T	Gaillard 2011 [47]	Cross Sectional Study	molecular techniques	349	solid/liquid	SD Bioline
Gaillard, T	Gaillard 2011 [47]	Cross Sectional Study	molecular techniques	349	solid/liquid	BD MGIT TBcID
Lu, P	Lu 2011 [48]	Cross Sectional Study	immunochromatographic assay	291	Löwenstein–Jensen medium/liquid	BD MGIT TBcID
Said, H	Said 2011 [49]	Cross Sectional Study	molecular assays	225	liquid	BD MGIT TBcID
Toihir, A	Toihir 2011 [50]	Cross Sectional Study	standard biochemical detection	171	Löwenstein–Jensen medium	SD Bioline
Muyoyeta, M	Muyoyeta 2010 [51]	Cross Sectional Study	phenotypic, biochemical, and molecular techniques.	623	solid/liquid	Capilia TB
Hillemann, D	Hillemann 2005 [52]	Cross Sectional Study	Molecular method	172	Liquid/Solid	Capilia TB
Wang, J	Wang 2007 [53]	Cross Sectional Study	Biochemistry method/Culturing	242	Liquid	Capilia TB
Ismail, N	Ismail 2009 [54]	Cross Sectional Study	Biochemistry method/Culturing	96	Liquid	SD Bioline
Ngamlert K	Ngamlert 2009 [55]	Cross Sectional Study	Biochemistry method/Culturing	247	Liquid	Capilia TB
Shen, G	Shen 2009 [56]	Cross Sectional Study	Biochemistry method/Culturing	233	Liquid	Capilia TB
Chihota, V	Chihota 2010 [57]	Cross Sectional Study	Biochemistry method	340	Liquid/Solid	Capilia TB

*CRS* Composite reference standard, *MTC Mycobacterium tuberculosis* complex, *PNB* ParaNitrobenzoic Acid

^a^328 were serum samples, 160 from patients with definite pulmonary tuberculosis

### Overall accuracy of MPT64

**Fig. 3:**
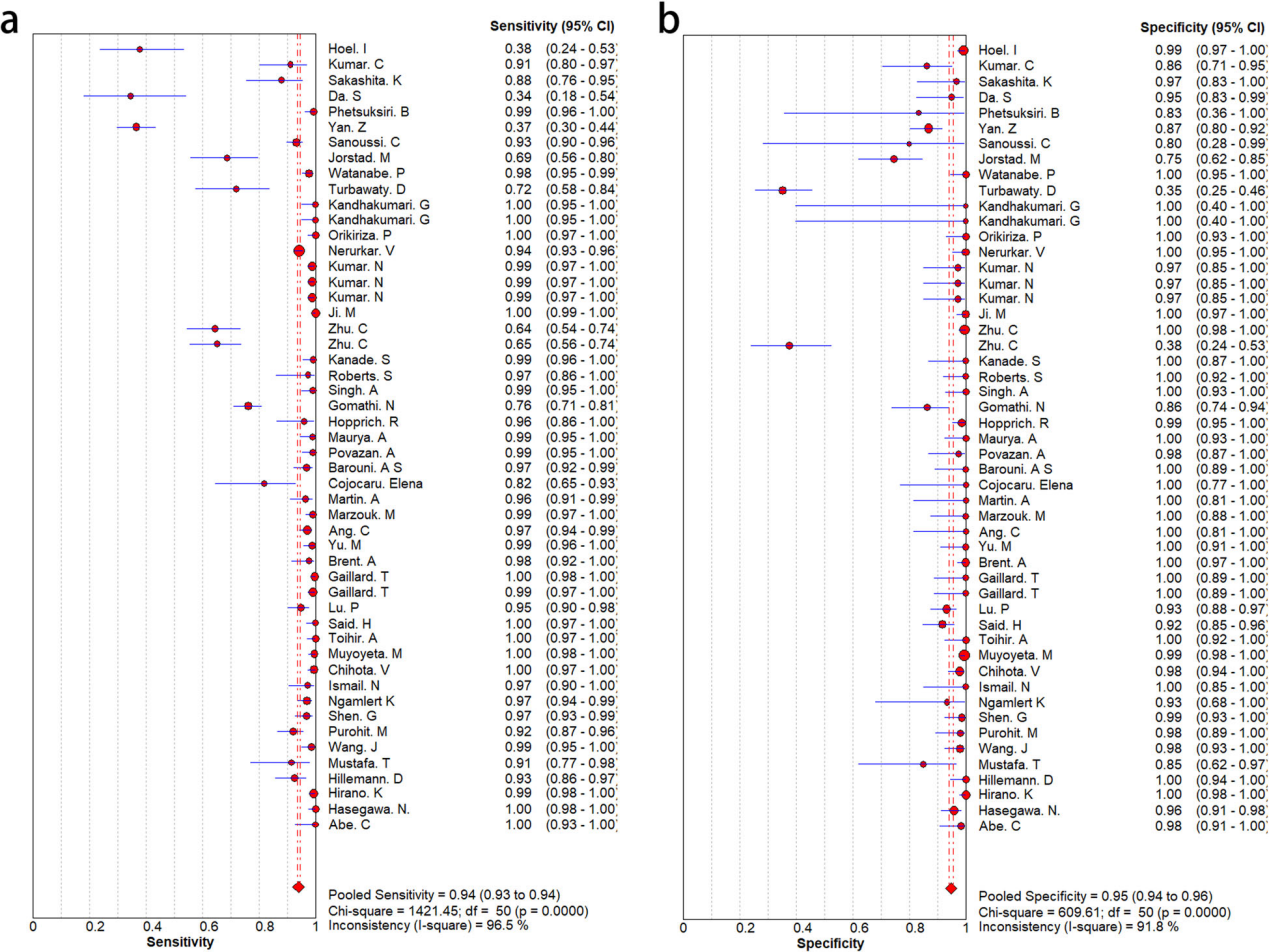
Forest plots of sensitivity and specificity. **a**. sensitivity, **b**. specificity

**Fig. 4:**
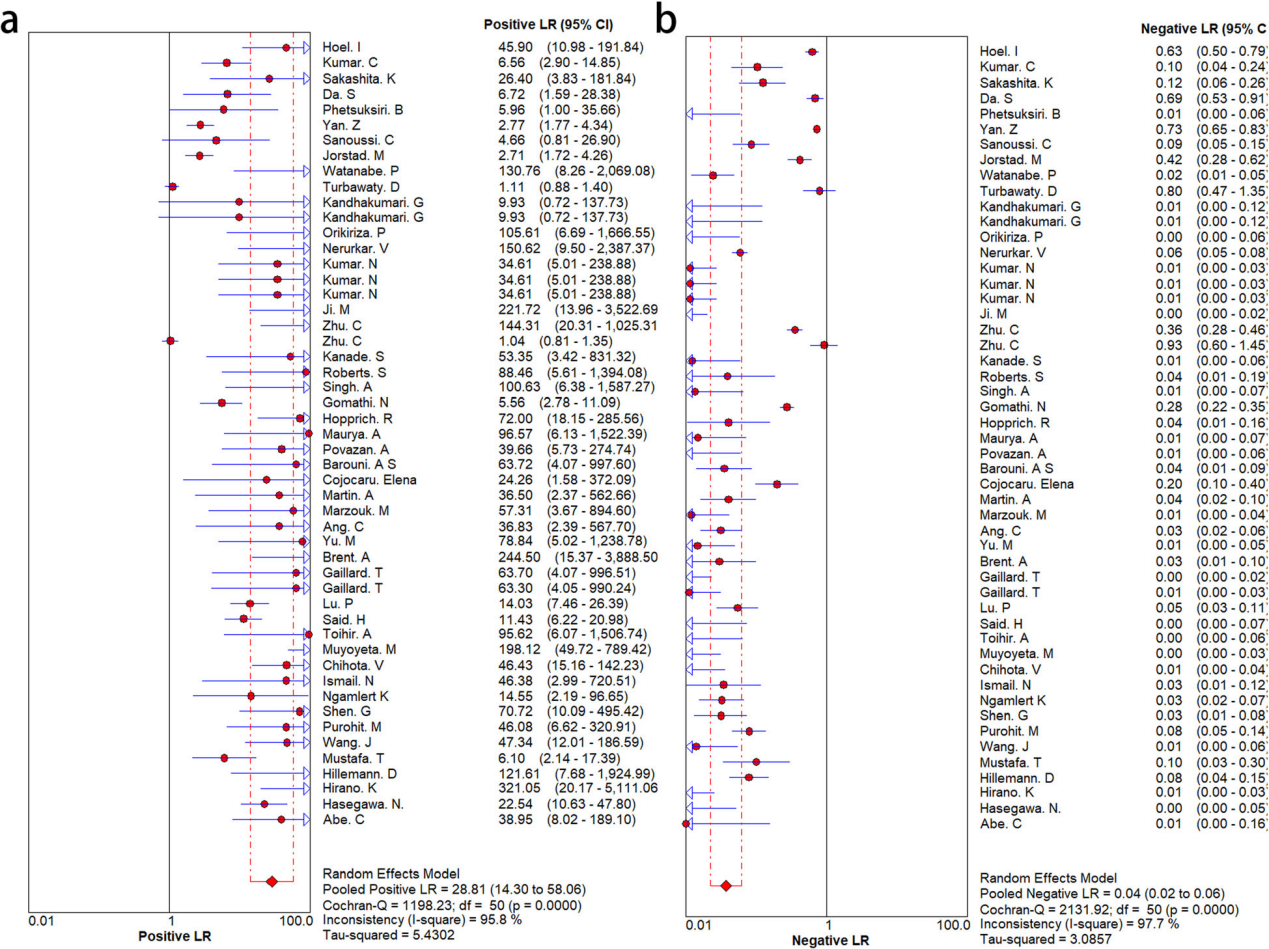
Forest plots of positive LR and negative LR. **a**. positive LR, **b**. negative LR

**Fig. 5:**
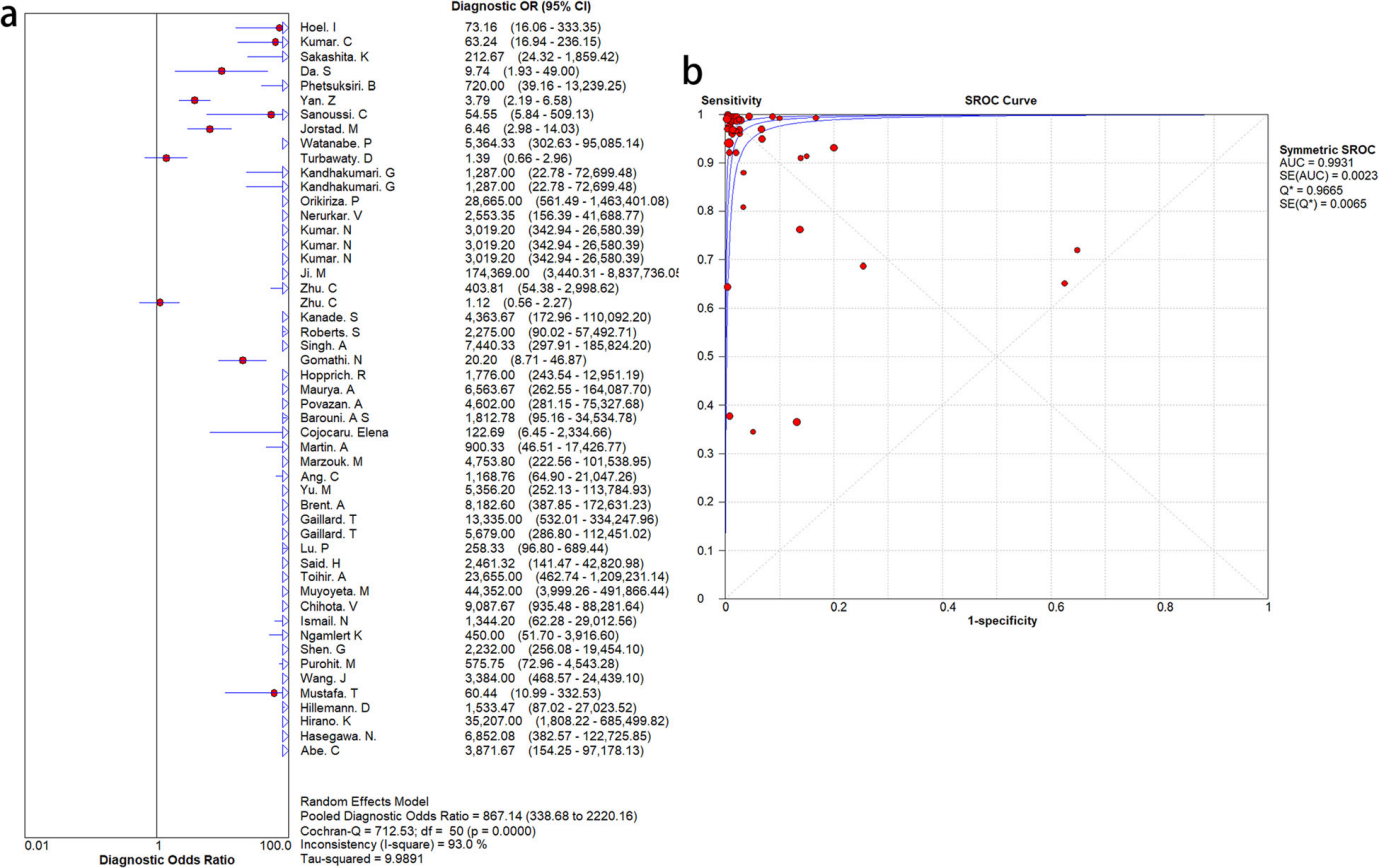
Overall diagnostic efficacy of MPT64 assays for *Mycobacterium tuberculosis*. **a**. diagnostic OR for the diagnosis of *Mycobacterium tuberculosis* infection, **b**. SROC curve

To explore the diagnostic accuracy of MPT64 for MTB, we adopted a random-effects model. MPT64 showed good diagnostic performance for MTB. However, there was obvious heterogeneity among the 46 studies. The SEN and SPE and associated 95% CIs were 0.92 (0.91–0.93) and 0.95 (0.94–0.95), respectively (Fig. 3). The NLR and PLR were 0.04 (95% CI 0.03–0.07) and 25.32 (95% CI 12.38–51.78), respectively (Fig. 4). DOR was 639.60 (243.04–1683.18) (Fig. 5). The AUC was 0.99 (Fig. 5), indicating that the diagnostic accuracy of the MPT64 test was very high. The result of overall accuracy of MPT64 was shown in Table 2.

**Table 2:** Overall Accuracy of MPT64

SEN	SPE	NLR	PLR	DOR
0.92 (95% CI 0.91–0.93)	0.95 (95% CI 0.94–0.95)	0.04 (95% CI 0.03–0.07)	25.32 (95% CI 12.38–51.78)	639.60 (95% CI 243.04–1683.18)

*SEN* Sensitivity, *SPE* Specificity, *NLR* Negative likelihood ratio, *PLR* Positive likelihood ratio, *DOR* Diagnostic odds ratio

**Fig. 6:**
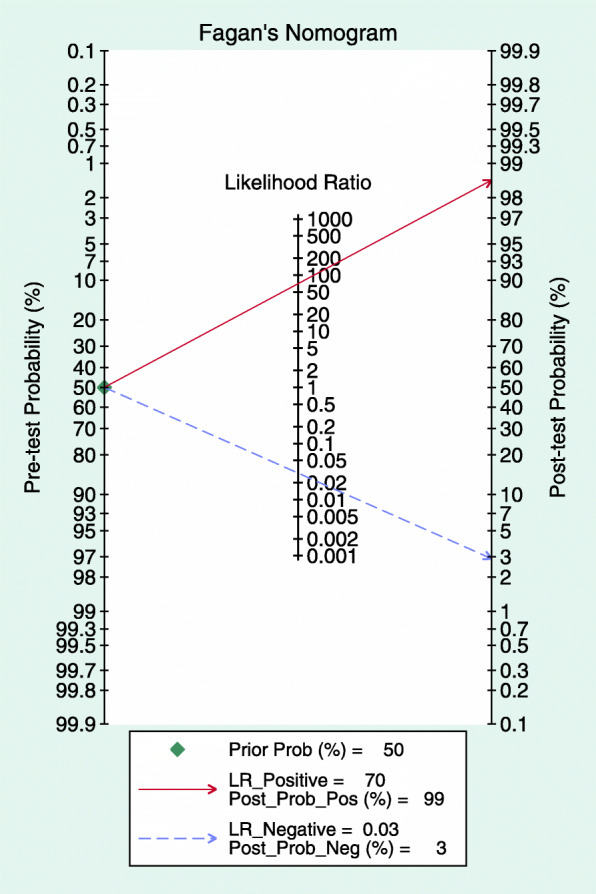
Fagan plot of disease probabilities based on Bayes’ theorem

According to the Fagan plot (Fig. 6), the pre-test probability was 50% and the post-test probability was 99%. The post-test probability significantly improved.

### Subgroup analysis of the three commercial kits

**Fig. 7:**
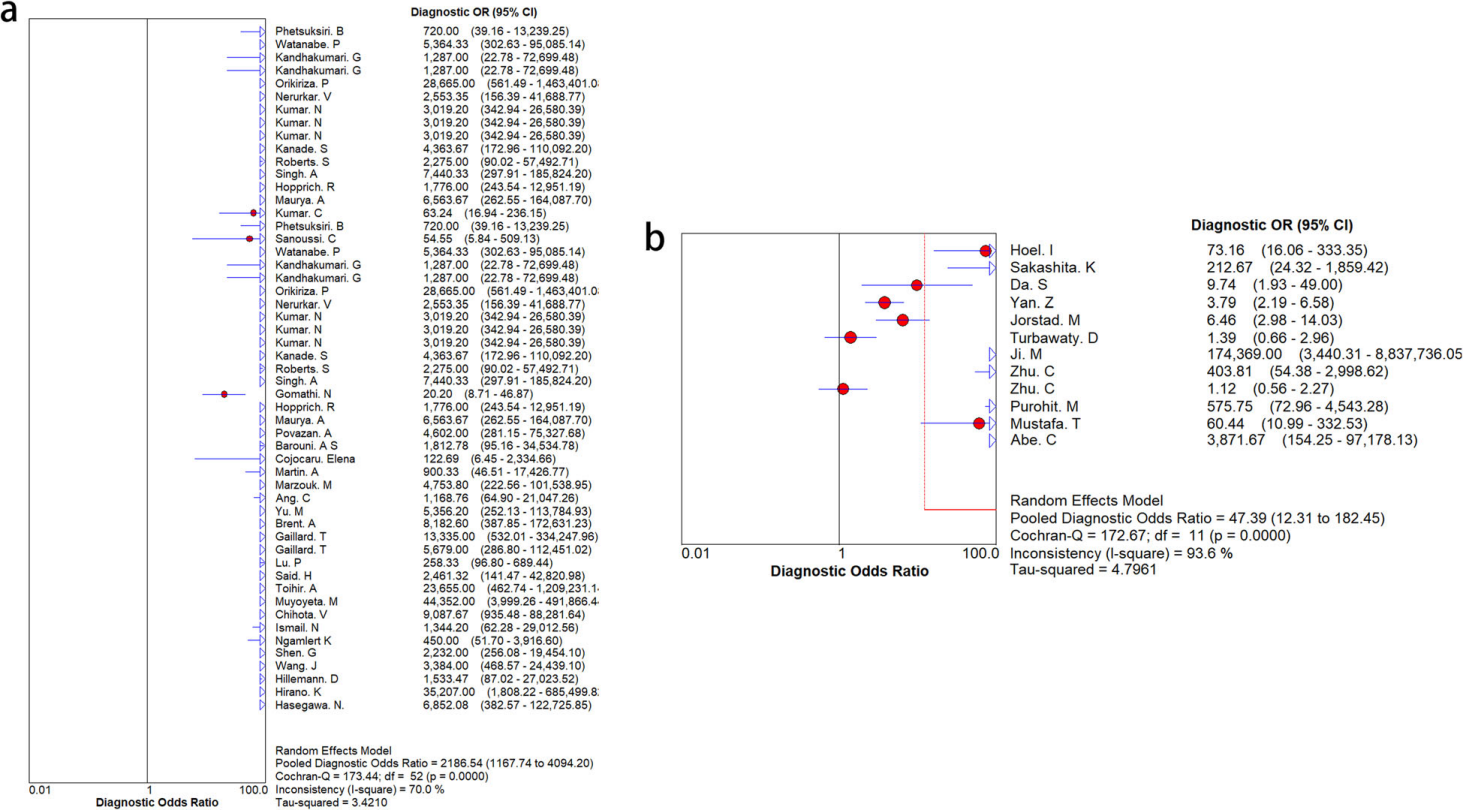
The results of subgroup analysis between “three commercial kits group” and other detection methods. **a**. the result of “three commercial kits group”, **b**. the result of other detection methods group

**Fig. 8:**
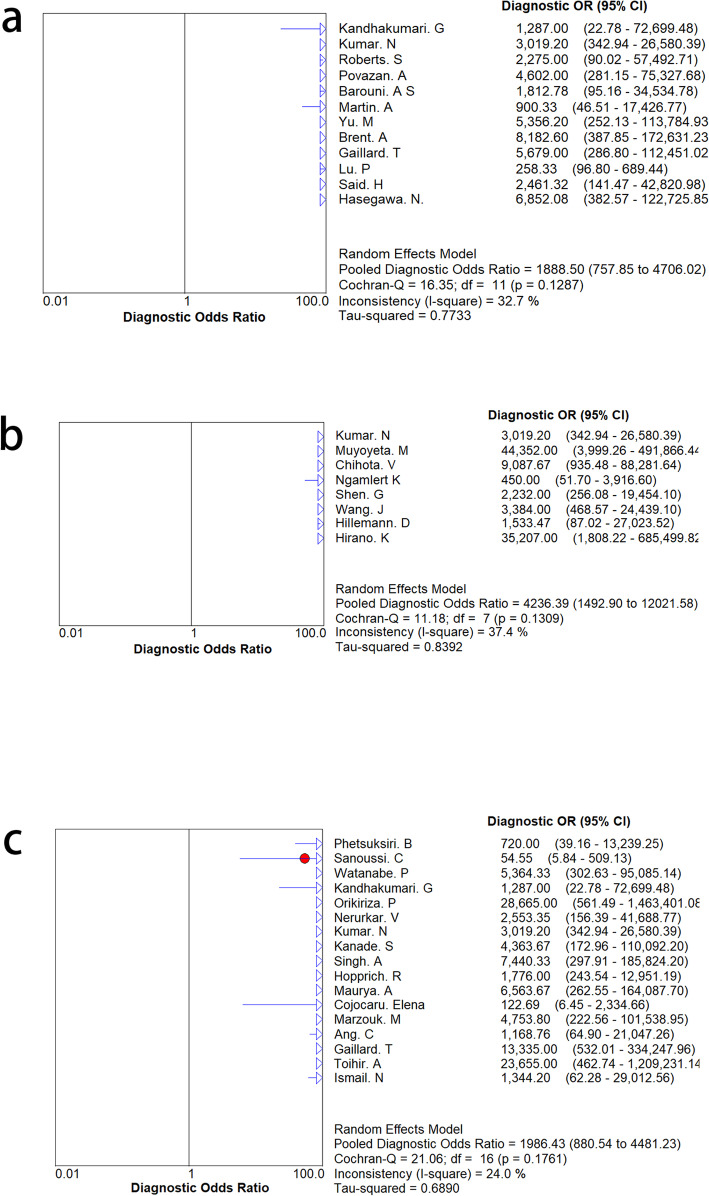
The results of subgroup analysis for the three commercial kits. **a**. the result of BD MGIT TBcID kit, **b**. the result of Capilia TB kit, **c**. the result of SD Bioline kit

The results of the subgroup analyses of the three kits are shown in Table 3, Fig. 7 and Fig. 8. SD Bioline had high pooled specificity and sensitivity for MPT64 detection. There was no significant change in SEN and SPE, indicating that the accuracy of the diagnosis did not depend on the kit.

**Table 3:** Subgroup analyses for three commercial kits

Kit	SEN	SPE	SROC
BD MGIT TBcID	0.98 (0.98–0.99)	0.97 (0.95–0.98)	0.994
Capilia TB	0.98 (0.98–0.99)	0.99 (0.98–1.00)	0.9969
SD Bioline	0.97 (0.96–0.97)	0.99 (0.98–1.00)	0.9966

*SEN* Sensitivity, *SPE* Specificity

### Heterogeneity and publication Bias

**Fig. 9:**
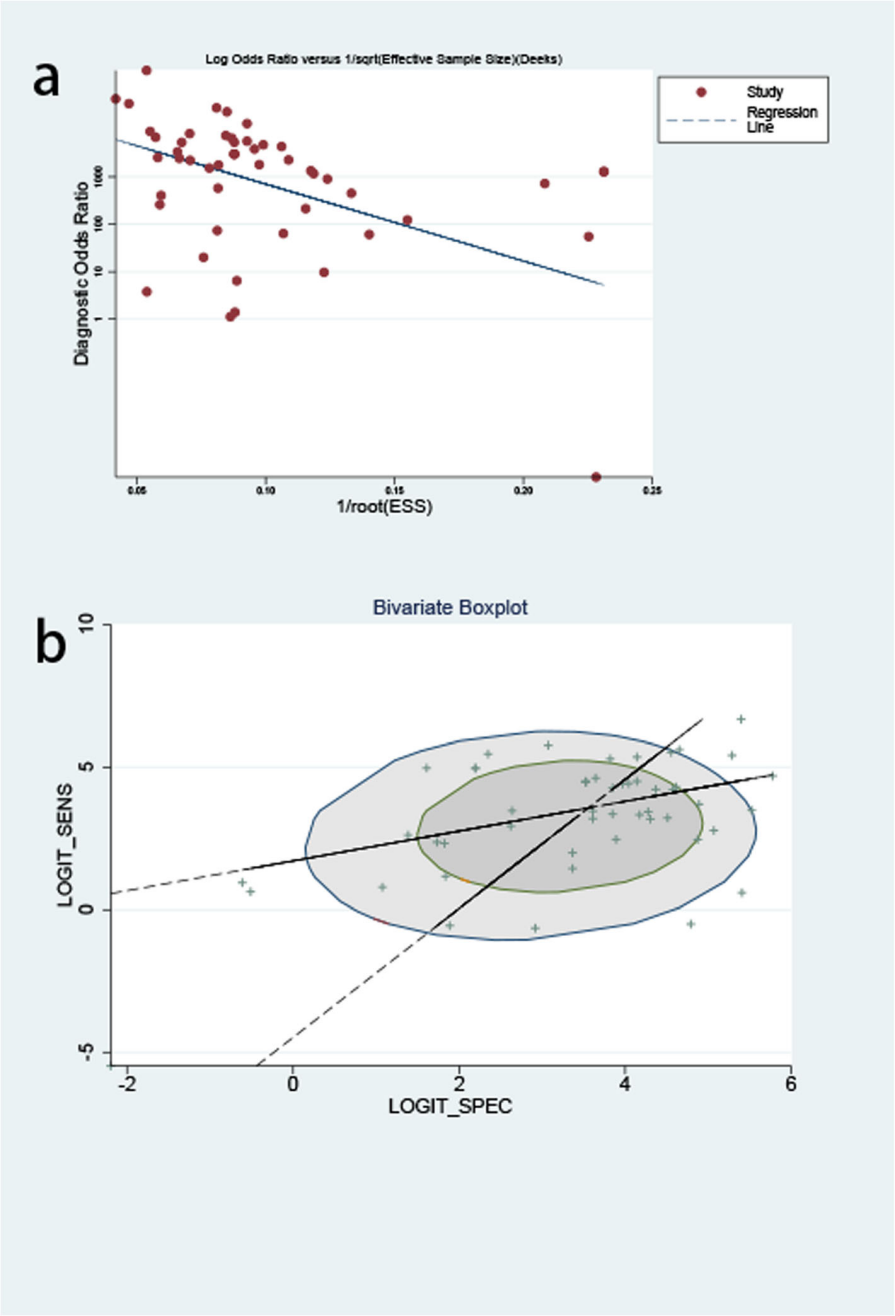
Publication bias for MPT64 detection for MTB. **a**. Deeks’ funnel plot asymmetry test to assess the publication bias for MPT64 detection for MTB; **b**. Bivariate boxplot

As shown by the results of subgroup analyses, the heterogeneity of “the three-kits group” was high. However, when we reviewed the full text and eliminated the research of Kumar et al. and Gomathi et al., the heterogeneity was significantly reduced (less than 50%). According to the bivariate boxplot (Fig. 9b), there were seven sets of data outside the circle, which also showed that there was significant heterogeneity in the overall research.

As shown in Fig. 9a, publication bias existed, with a *p* value of 0.012.

## Discussion

TB is a serious infectious disease and every year, millions of people worldwide contract MTB. Moreover, a large number of people die from TB [1]. Thus, there is an urgent and essential need to develop real-time, portable, and sensitive techniques to detect MTB and its drug-resistant mutations. This study evaluated the accuracy of the diagnosis of MTB by using various MPT64-detecting methods.

Although Yin et al [12] conducted similar research in 2013, new articles have been published since then. Therefore, we have updated their research. Our study analyzed more articles than theirs, which included only 28 articles. Therefore, for now, our research is more comprehensive. Moreover, we added a Fagan plot, which verified the clinical application value of MPT64. After using the MPT64 test, the post-test probability significantly improved. Moreover, when analyzing the heterogeneity, we came to the opposite conclusion as Yin et al. Their research showed that except for the comprehensive sensitivity of the MGIT TBc ID test and the pooled specificity of the SD Bioline Ag MPT64 rapid determination, all statistical indicators had considerable heterogeneity. However, our research found that after excluding the two articles that had problems in sample handling, there was no significant heterogeneity (I^2^ < 50%) between the three commercial kits.

The overall result showed that MPT64 had a good test performance. In the subgroup analyses, we eliminated two articles because one article mixed weak positives with positives and the samples of another article were partially contaminated. Finally, the results of the subgroup analyses showed that the diagnostic accuracy of MPT64 did not depend on the kit. In addition, there was no obvious heterogeneity between the three commercial kits. Therefore, when resources are insufficient, cheaper kits can be used.

In our study, we only analyzed the impact of the kit on the diagnostic accuracy and did not analyze whether other factors, such as sample type, affect it. In addition, the diagnostic efficacy of MPT64 for different types of tuberculosis is worth investigating. The diagnosis of MPT64 in different populations remains to be studied. For instance, Jorstad et al [21] analyzed the influence of age on diagnostic accuracy and found that the sensitivity of the MPT64 test was significantly higher in children than in adults. Due to insufficient extracted data, we were unable to analyze and verify this.

## Conclusion

In conclusion, the MPT64 test shows a good diagnostic performance for MTB; it has high sensitivity and specificity as well as clinical application value. Moreover, the three commercial kits, SD Bioline, Capilia TB, and BD MGIT TBcID, are not heterogeneous. Therefore, when resources are insufficient, cheaper kits can be used.

## Supplementary Information

**Additional file 1: Table S1.** Subgroup analysis of reference standard.

**Additional file 2.**

## Data Availability

The datasets used and/or analyzed during the current study are available from the corresponding author on reasonable request.

## References

[1] WHO (2019). Global tuberculosis report 2019.

[2] Golden MP, Vikram HR (2005). Extrapulmonary tuberculosis: an overview. Am Fam Physician.

[3] Drain PK (2018). Incipient and subclinical tuberculosis: a clinical review of early stages and progression of infection. Clin Microbiol Rev.

[4] Khan MK, Islam MN, Ferdous J, Alam MM (2019). An overview on epidemiology of tuberculosis. Mymensingh Med J.

[5] Gupta S, Kakkar V (2018). Recent technological advancements in tuberculosis diagnostics - a review. Biosens Bioelectron.

[6] Mustafa AS, Shaban F (2010). Mapping of Th1-cell epitope regions of mycobacterium tuberculosis protein MPT64 (Rv1980c) using synthetic peptides and T-cell lines from M. tuberculosis-infected healthy humans. Med Princ Pract.

[7] Xiao T, Jiang Y, Li G, Pang H, Zhao L, Zhao X, Wan K (2019). Polymorphism of MPT64 and PstS1 in mycobacterium tuberculosis is not likely to affect relative immune reaction in human. Medicine (Baltimore).

[8] Kumar N, Agarwal A, Dhole TN, Sharma YK (2015). Rapid identification of mycobacterium tuberculosis complex in clinical isolates by combining presumptive cord formation and MPT64 antigen Immunochromatographic assay. Indian J Tuberc.

[9] Sakashita K, Takeuchi R, Takeda K, Takamori M, Ito K, Igarashi Y, Hayashi E, Iguchi M, Ono M, Kashiyama T, Tachibana M, Miyakoshi J, Yano K, Sato Y, Yamamoto M, Murata K, Wada A, Chikamatsu K, Aono A, Takaki A, Nagai H, Yamane A, Kawashima M, Komatsu M, Nakaishi K, Watabe S, Mitarai S (2020). Ultrasensitive enzyme-linked immunosorbent assay for the detection of MPT64 secretory antigen to evaluate mycobacterium tuberculosis viability in sputum. Int J Infect Dis.

[10] Phetsuksiri B, Rudeeaneksin J, Srisungngam S, Bunchoo S, Klayut W, Sangkitporn S, Nakajima C, Hamada S, Suzuki Y (2019). Loop-mediated isothermal amplification for rapid identification of mycobacterium tuberculosis in comparison with Immunochromatographic SD bioline MPT64 rapid (®) in a high burden setting. Jpn J Infect Dis.

[11] Gomathi NS, Devi SM, Lakshmi R, Ramachandran R, Wares DF, Kumar V, Selvakumar N (2012). Capilia test for identification of mycobacterium tuberculosis in MGIT-positive cultures. Int J Tuberc Lung Dis.

[12] Yin X (2013). Commercial MPT64-based tests for rapid identification of mycobacterium tuberculosis complex: a meta-analysis. J Inf Secur.

[13] Whiting PF, Rutjes AW, Westwood ME, Mallett S, Deeks JJ, Reitsma JB, Leeflang MM, Sterne JA, Bossuyt PM, QUADAS-2 Group (2011). QUADAS-2: a revised tool for the quality assessment of diagnostic accuracy studies. Ann Intern Med.

[14] Yang WT, Parikh JR, Stavros AT, Otto P, Maislin G (2018). Exploring the negative likelihood ratio and how it can be used to minimize false-positives in breast imaging. AJR Am J Roentgenol.

[15] Glas AS, Lijmer JG, Prins MH, Bonsel GJ, Bossuyt PM (2003). The diagnosticodds ratio: a single indicator of test performance. J Clin Epidemiol.

[16] Hoel IM, Sviland L, Syre H, Dyrhol-Riise AM, Skarstein I, Jebsen P, Jørstad MD, Wiker H, Mustafa T (2020). Diagnosis of extrapulmonary tuberculosis using the MPT64 antigen detection test in a high-income low tuberculosis prevalence setting. BMC Infect Dis.

[17] Kumar C (2020). The MPB64 immunochromatography assay: an analysis of doubtful results. Trop Dr.

[18] Da SR (2019). IgA and IgG antibody detection of mycobacterial antigens in pleural fluid and serum from pleural tuberculous patients. BMC Immunol.

[19] Yan ZH, Yi L, Wei PJ, Jia HY, Wang J, Wang XJ, Yang B, Gao X, Zhao YL, Zhang HT (2018). Evaluation of panels of mycobacterium tuberculosis antigens for serodiagnosis of tuberculosis. Int J Tuberc Lung Dis.

[20] Sanoussi CN (2018). Low sensitivity of the MPT64 identification test to detect lineage 5 of the mycobacterium tuberculosis complex. J Med Microbiol.

[21] Jorstad MD (2018). MPT64 antigen detection test improves routine diagnosis of extrapulmonary tuberculosis in a low-resource setting: a study from the tertiary care hospital in Zanzibar. PLoS One.

[22] Watanabe PJ (2018). Use of an immunochromatographic assay for rapid identification of mycobacterium tuberculosis complex clinical isolates in routine diagnosis. J Med Microbiol.

[23] Turbawaty DK (2017). Comparison of the performance of urinary mycobacterium tuberculosis antigens cocktail (ESAT6, CFP10, and MPT64) with culture and microscopy in pulmonary tuberculosis patients. Int J Microbiol.

[24] Kandhakumari G, Stephen S (2017). Evaluation of a new rapid kit, BD MGIT TBc identification test for confirmation of mycobacterium tuberculosis complex. Indian J Pathol Microbiol.

[25] Orikiriza P, Nyehangane D, Atwine D, Kisakye JJ, Kassaza K, Amumpaire JM, Boum Y (2017). Evaluation of the SD bioline TB Ag MPT64 test for identification of mycobacterium tuberculosis complex from liquid cultures in southwestern Uganda. Afr J Lab Med.

[26] Nerurkar V, Kattungal S, Bhatia S (2016). Utility of MPT64 antigen test for differentiating mycobacteria: can correlation with liquid culture smear morphology add further value?. Indian J Pathol Microbiol.

[27] Ji M, Cho B, Cho YS, Park SY, Cho SN, Jeon BY, Yoon BS (2014). Development of a quantitative sandwich enzyme-linked immunosorbent assay for detecting the MPT64 antigen of mycobacterium tuberculosis. Yonsei Med J.

[28] Zhu C (2013). Correction: evaluation of the clinical value of ELISA based on MPT64 antibody aptamer for serological diagnosis of pulmonary tuberculosis. BMC Infect Dis.

[29] Hopprich R, Shephard L, Taing B, Kralj S, Smith A, Lumb R (2012). Evaluation of (SD) MPT64 antigen rapid test, for fast and accurate identification of mycobacterium tuberculosis complex. Pathology.

[30] Kanade S, Nataraj G, Suryawanshi R, Mehta P (2012). Utility of MPT 64 antigen detection assay for rapid characterization of mycobacteria in a resource constrained setting. Indian J Tuberc.

[31] Roberts SA, Lowe O, Pandey S, Williamson DA, Newton S, Vaughan R (2012). Comparison of the MGIT TBc immunochromatographic assay with the Accuprobe gen-probe TB assay for identification of mycobacterium tuberculosis complex: results from a low-burden tuberculosis setting. Diagn Microbiol Infect Dis.

[32] Singh AK (2012). Evaluation of rapid TB antigen MPT64 test for identification of mycobacterium tuberculosis complex in liquid culture isolates at tertiary care center in northern India. Int J Infect Dis.

[33] Martin A, Bombeeck D, Mulders W, Fissette K, de Rijk P, Palomino JC (2011). Evaluation of the TB Ag MPT64 rapid test for the identification of mycobacterium tuberculosis complex. Int J Tuberc Lung Dis.

[34] Marzouk M, Kahla IB, Hannachi N, Ferjeni A, Salma WB, Ghezal S, Boukadida J (2011). Evaluation of an immunochromatographic assay for rapid identification of mycobacterium tuberculosis complex in clinical isolates. Diagn Microbiol Infect Dis.

[35] Ang CF, Cajucom MAM, Kim Y, Bang H, Lee H, Cho SN, Montalban CS (2011). Evaluation of a rapid assay for identification of mycobacterium tuberculosis grown in solid and liquid media. Int J Tuberc Lung Dis.

[36] Yu MC, Chen HY, Wu MH, Huang WL, Kuo YM, Yu FL, Jou R (2011). Evaluation of the rapid MGIT TBc identification test for culture confirmation of mycobacterium tuberculosis complex strain detection. J Clin Microbiol.

[37] Purohit MR, Mustafa T, Wiker HG, Mørkve O, Sviland L (2007). Immunohistochemical diagnosis of abdominal and lymph node tuberculosis by detecting mycobacterium tuberculosis complex specific antigen MPT64. Diagn Pathol.

[38] Mustafa T, Wiker HG, Mfinanga SGM, Mørkve O, Sviland L (2006). Immunohistochemistry using a mycobacterium tuberculosis complex specific antibody for improved diagnosis of tuberculous lymphadenitis. Mod Pathol.

[39] Hirano K, Aono A, Takahashi M, Abe C (2004). Mutations including IS6110 insertion in the gene encoding the MPB64 protein of Capilia TB-negative mycobacterium tuberculosis isolates. J Clin Microbiol.

[40] Hasegawa N, Miura T, Ishii K, Yamaguchi K, Lindner TH, Merritt S, Matthews JD, Siddiqi SH (2002). New simple and rapid test for culture confirmation of mycobacterium tuberculosis complex: a multicenter study. J Clin Microbiol.

[41] Abe C, Hirano K, Tomiyama T (1999). Simple and rapid identification of the mycobacterium tuberculosis complex by immunochromatographic assay using anti-MPB64 monoclonal antibodies. J Clin Microbiol.

[42] Maurya AK, Nag VL, Kant S, Kushwaha RA, Kumar M, Mishra V, Rahman W, Dhole TN (2012). Evaluation of an immunochromatographic test for discrimination between mycobacterium tuberculosis complex & non tuberculous mycobacteria in clinical isolates from extra-pulmonary tuberculosis. Indian J Med Res.

[43] Povazan A (2012). Use of immunochromatographic assay for rapid identification of mycobacterium tuberculosis complex from liquid culture. Bosn J Basic Med Sci.

[44] Barouni AS (2012). Evaluation of the BD MGIT (TM) TBc identification test for rapid identification of mycobacterium tuberculosis complex from positive BACTEC MGIT 960 cultures in a routine laboratory work. Afr J Microbiol Res.

[45] Cojocaru E (2012). Identification mycobacterium tuberculosis complex using an immunochromatographic test running title: a useful test for M. tuberculosis identification. Romanian Biotechnol Lett.

[46] Brent AJ, Mugo D, Musyimi R, Mutiso A, Morpeth S, Levin M, Scott JAG (2011). Performance of the MGIT TBc identification test and meta-analysis of MPT64 assays for identification of the mycobacterium tuberculosis complex in liquid culture. J Clin Microbiol.

[47] Gaillard T, Fabre M, Martinaud C, Vong R, Brisou P, Soler C (2011). Assessment of the SD bioline Ag MPT64 rapid™ and the MGIT™ TBc identification tests for the diagnosis of tuberculosis. Diagn Microbiol Infect Dis.

[48] Lu PL, Yang YC, Huang SC, Jenh YS, Lin YC, Huang HH, Chang TC (2011). Evaluation of the Bactec MGIT 960 system in combination with the MGIT TBc identification test for detection of mycobacterium tuberculosis complex in respiratory specimens. J Clin Microbiol.

[49] Said HM, Ismail N, Osman A, Velsman C, Hoosen AA (2011). Evaluation of TBc identification immunochromatographic assay for rapid identification of mycobacterium tuberculosis complex in samples from broth cultures. J Clin Microbiol.

[50] Toihir AH (2011). Validation of an immunochromatographic assay kit for the identification of the mycobacterium tuberculosis complex. Mem Inst Oswaldo Cruz.

[51] Muyoyeta M, de Haas PEW, Mueller DH, van Helden PD, Mwenge L, Schaap A, Kruger C, Gey van Pittius NC, Lawrence K, Beyers N, Godfrey-Faussett P, Ayles H (2010). Evaluation of the Capilia TB assay for culture confirmation of mycobacterium tuberculosis infections in Zambia and South Africa. J Clin Microbiol.

[52] Hillemann D, Rusch-Gerdes S, Richter E (2005). Application of the Capilia TB assay for culture confirmation of mycobacterium tuberculosis complex isolates. Int J Tuberc Lung Dis.

[53] Wang JY, Lee LN, Lai HC, Hsu HL, Jan IS, Yu CJ, Hsueh PR, Yang PC (2007). Performance assessment of the Capilia TB assay and the BD ProbeTec ET system for rapid culture confirmation of mycobacterium tuberculosis. Diagn Microbiol Infect Dis.

[54] Ismail NA, Baba K, Pombo D, Hoosen AA (2009). Use of an immunochromatographic kit for the rapid detection of mycobacterium tuberculosis from broth cultures. Int J Tuberc Lung Dis.

[55] Ngamlert K, Sinthuwattanawibool C, McCarthy KD, Sohn H, Starks A, Kanjanamongkolsiri P, Anek-vorapong R, Tasaneeyapan T, Monkongdee P, Diem L, Varma JK (2009). Diagnostic performance and costs of Capilia TB for mycobacterium tuberculosis complex identification from broth-based culture in Bangkok. Thailand Trop Med Int Health.

[56] Shen GH, Chen CH, Hung CH, Wu KM, Lin CF, Sun YW, Chen JH (2009). Combining the Capilia TB assay with smear morphology for the identification of mycobacterium tuberculosis complex. Int J Tuberc Lung Dis.

[57] Chihota VN (2010). Liquid vs. solid culture for tuberculosis: performance and cost in a resource-constrained setting. Int J Tuberc Lung Dis.

