# Supplementary material for: MPT64 assays for the rapid detection of Mycobacterium tuberculosis
Source: BMC Infect Dis. 2021 Apr 10;21:336. doi: 10.1186/s12879-021-06022-w (PMC8035777; doi:10.1186/s12879-021-06022-w)
Supplement: Supplementary file 1 — Additional file 1: Table S1. Subgroup analysis of reference standard. [file 12879_2021_6022_MOESM1_ESM.pdf]

Table 1. Characteristics of the studies included in the articles.

Author	Study	Study Design	Reference Test	Sample size	Medium	Method of detection
Hoel, I	Hoel 2020[14]	Cross Sectional Study	composite reference standard (CRS)	288	liquid	ICC Staining (Dako Envision + System-HRP kit)
Kumar, C	Kumar2020[15]	Cross Sectional Study	Duplex PCR assay	92	liquid	BD MGIT TBcID
Sakashita, K	Sakashita2020[9]	Cross Sectional Study	bacteriologically diagnosed	80	solid	ELISA
Da, S	Da 2019[16]	Cross Sectional Study	CRS	68	liquid	ELISA
Phetsuksiri, B	Phetsuksiri 2019[10]	Cross Sectional Study	Culture followed by identification of MTC	151	liquid	SD Bioline
Yan, Z	Yan 2018[17]	Cross Sectional Study	CRS	352	unclear	BD OptEIAe Reagent Set B ELISA kit
Sanoussi, C	Sanoussi2018[18]	Cross Sectional Study	spoligotyping or PNB/catalase	327	solid	SD Bioline
Jorstad, M	Jorstad 2018[19]	Cross Sectional Study	CRS	126	Löwenstein–Jensen medium	t 1/250 dilution and Dako kit
Watanabe, P	Watanabe 2018[20]	Cross Sectional Study	phenotypic techniques and molecular tests（such as conventional or real-time PCR, line probe assays and in-house (PCR and restriction-enzyme analysis) PRA-hsp65 molecular assay)	375	liquid/solid	SD Bioline
Turbawaty, D	Turbawaty 2017[21]	Cross Sectional Study	acid-fast bacilli and mycobacterial culture	141	liquid	ICT
Kandhakumari, G	Kandhakumari 2017[22]	Cross Sectional Study	Biochemistry method	75	solid	BD MGIT TBcID
Kandhakumari, G	Kandhakumari 2017[22]	Cross Sectional Study	Biochemistry method	75	solid	SD Bioline
Orikiriza, P	Orikiriza 2017[23]	Cross Sectional Study	Biochemistry method/Culturing of mycobacteria	188	liquid	SD Bioline
Nerurkar, V	Nerurkar 2016[24]	Cross Sectional Study	Culturing of mycobacteria	1093	liquid	SD Bioline
Kumar, N	Kumar 2015[8]	Cross Sectional Study	Biochemistry method/Molecular method (PNB inhibition test)	484	Solid/liquid	SD Bioline/BD MGIT/Capilia TB
Ji, M	Ji 2014[25]	Cross Sectional Study	Culturing of mycobacteria	504	liquid	ELISA
Zhu, C*	Zhu 2013[26]	Cross Sectional Study	Biochemistry method/Culturing	328	solid	ELISA
Zhu, C*	Zhu 2013[26]	Cross Sectional Study	Biochemistry method/Culturing	160	solid	ELISA
Hopprich, R	Hopprich 2012[27]	Cross Sectional Study	Molecular method +Biochemistry method	200	liquid	SD Bioline
Kanade, S	Kanade 2012[28]	Cross Sectional Study	molecular method	150	solid	SD Bioline
Roberts, S	Roberts 2012[29]	Cross Sectional Study	molecular method	83	liquid	BD MGIT TBcID
Singh, A	Singh 2012[30]	Cross Sectional Study	Culturing	161	liquid	SD Bioline
Martin, A	Martin 2011[31]	Cross Sectional Study	molecular method	131	liquid	BD MGIT TBcID
Marzouk, M	Marzouk 2011[32]	Cross Sectional Study	Biochemistry method/Culturing	238	Solid/liquid	SD Bioline
Ang, C	Ang 2011[33]	Cross Sectional Study	Biochemistry method/Culturing	294	Solid/liquid	SD Bioline
Yu, M	Yu 2011[34]	Cross Sectional Study	Biochemistry method/Culturing	210	liquid	BD MGIT TBcID
Purohit, M	Purohit 2007[35]	Cross Sectional Study	molecular method	203	solid	DakoCytomation

Mustafa, T	Mustafa 2006[36]	Cross Sectional Study	molecular method	55	liquid	NA
Hirano, K	Hirano 2004[37]	Cross Sectional Study	molecular method	545	liquid	Capilia TB
Hasegawa, N.	Hasegawa 2002[38]	Cross Sectional Study	molecular method or Biochemistry method	304	liquid	BD MGIT TBcID
Abe, C	Abe 1999[39]	Cross Sectional Study	molecular method	108	liquid	NA
Gomathi, N	Gomathi 2012[11]	Cross Sectional Study	Biochemistry method	346	Liquid	Capilia TB
Maurya, A	Maurya 2012[40]	Cross Sectional Study	Biochemistry method	150	Liquid	SD Bioline
Povazan, A	Povazan 2012[41]	Cross Sectional Study	Biochemistry method	123	Liquid	BD MGIT TBcID
Barouni, A S	Barouni, A S 2012[42]	Cross Sectional Study	Biochemistry method	161	Liquid	BD MGIT TBcID
Cojocaru, Elena	Cojocaru 2012[43]	Cross Sectional Study	Biochemistry method	47	Liquid/Solid	SD Bioline
Brent, A	Brent 2011[44]	Cross Sectional Study	molecular method	208	liquid	BD MGIT TBcID
Gaillard, T	Gaillard 2011[45]	Cross Sectional Study	molecular techniques	349	solid/liquid	SD Bioline
Gaillard, T	Gaillard 2011[45]	Cross Sectional Study	molecular techniques	349	solid/liquid	BD MGIT TBcID
Lu, P	Lu 2011[46]	Cross Sectional Study	immunochromatographic assay	291	Löwenstein–Jensen medium/liquid	BD MGIT TBcID
Said, H	Said 2011[47]	Cross Sectional Study	molecular assays	225	liquid	BD MGIT TBcID
Toihir, A	Toihir 2011[48]	Cross Sectional Study	standard biochemical detection	171	Löwenstein–Jensen medium	SD Bioline
Muyoyeta, M	Muyoyeta 2010[49]	Cross Sectional Study	phenotypic, biochemical, and molecular techniques.	623	solid/liquid	Capilia TB
Hillemann, D	Hillemann 2005[50]	Cross Sectional Study	Molecular method	172	Liquid/Solid	Capilia TB
Wang, J	Wang 2007[51]	Cross Sectional Study	Biochemistry method/Culturing	242	Liquid	Capilia TB
Ismail, N	Ismail 2009[52]	Cross Sectional Study	Biochemistry method/Culturing	96	Liquid	SD Bioline
Ngamlert K	Ngamlert 2009[53]	Cross Sectional Study	Biochemistry method/Culturing	247	Liquid	Capilia TB
Shen, G	Shen 2009[54]	Cross Sectional Study	Biochemistry method/Culturing	233	Liquid	Capilia TB
Chihota, V	Chihota 2010[55]	Cross Sectional Study	Biochemistry method	340	Liquid/Solid	Capilia TB

CRS=composite reference standard; MTC=Mycobacterium tuberculosis complex; PNB=ParaNitrobenzoic Acid.

*: 328 were serum samples, 160 from patients with definite pulmonary tuberculosis.
