# Supplementary material for: MPT64 assays for the rapid detection of Mycobacterium tuberculosis
Source: BMC Infect Dis. 2021 Apr 10;21:336. doi: 10.1186/s12879-021-06022-w (PMC8035777; doi:10.1186/s12879-021-06022-w)
Supplement: Supplementary file 2 — Additional file 2. [file 12879_2021_6022_MOESM2_ESM.docx]

| Year | Author | Study | TP | FP | FN | TN |
| --- | --- | --- | --- | --- | --- | --- |
| 2020 | Hoel, I | Hoel 2020 | 17 | 2 | 28 | 241 |
| 2020 | Kumar, C | Kumar2020 | 51 | 5 | 5 | 31 |
| 2020 | Sakashita, K | Sakashita2020 | 44 | 1 | 6 | 29 |
| 2019 | Da, S | Da 2019 | 10 | 2 | 19 | 37 |
| 2019 | Phetsuksiri, B | Phetsuksiri 2019 | 144 | 1 | 1 | 5 |
| 2018 | Yan, Z | Yan 2018 | 73 | 20 | 127 | 132 |
| 2018 | Sanoussi, C | Sanoussi2018 | 300 | 1 | 22 | 4 |
| 2018 | Jorstad, M | Jorstad 2018 | 44 | 16 | 20 | 47 |
| 2018 | Watanabe, P | Watanabe 2018 | 302 | 0 | 7 | 66 |
| 2017 | Turbawaty, D | Turbawaty 2017 | 36 | 59 | 14 | 32 |
| 2017 | Kandhakumari, G | Kandhakumari 2017 | 71 | 0 | 0 | 4 |
| 2017 | Kandhakumari, G | Kandhakumari 2017 | 71 | 0 | 0 | 4 |
| 2017 | Orikiriza, P | Orikiriza 2017 | 136 | 0 | 0 | 52 |
| 2016 | Nerurkar, V | Nerurkar 2016 | 955 | 0 | 59 | 79 |
| 2015 | Kumar, N | Kumar 2015 | 444 | 1 | 5 | 34 |
| 2015 | Kumar, N | Kumar 2015 | 444 | 1 | 5 | 34 |
| 2015 | Kumar, N | Kumar 2015 | 444 | 1 | 5 | 34 |
| 2014 | Ji, M | Ji 2014 | 394 | 0 | 0 | 110 |
| 2013 | Zhu, C | Zhu 2013 | 67 | 1 | 37 | 223 |
| 2013 | Zhu, C | Zhu 2013 | 73 | 30 | 39 | 18 |
| 2012 | Kanade, S | Kanade 2012 | 123 | 0 | 1 | 26 |
| 2012 | Roberts, S | Roberts 2012 | 37 | 0 | 1 | 45 |
| 2012 | Singh, A | Singh 2012 | 110 | 0 | 1 | 50 |
| 2012 | Gomathi, N | Gomathi 2012 | 225 | 7 | 70 | 44 |
| 2012 | Hopprich, R | Hopprich 2012 | 48 | 2 | 2 | 148 |
| 2012 | Maurya, A | Maurya 2012 | 101 | 0 | 1 | 48 |
| 2012 | Povazan, A | Povazan 2012 | 118 | 1 | 1 | 39 |
| 2012 | Barouni, A S | Barouni, A S 2012 | 125 | 0 | 4 | 32 |
| 2012 | Cojocaru, Elena | Cojocaru, Elena 2012 | 27 | 0 | 6 | 14 |
| 2011 | Martin, A | Martin 2011 | 109 | 0 | 4 | 18 |
| 2011 | Marzouk, M | Marzouk 2011 | 208 | 0 | 2 | 28 |
| 2011 | Ang, C | Ang 2011 | 268 | 0 | 8 | 18 |
| 2011 | Yu, M | Yu 2011 | 169 | 0 | 2 | 39 |
| 2011 | Brent, A | Brent 2011 | 81 | 0 | 2 | 125 |
| 2011 | Gaillard, T | Gaillard 2011 | 317 | 0 | 1 | 31 |
| 2011 | Gaillard, T | Gaillard 2011 | 315 | 0 | 3 | 31 |
| 2011 | Lu, P | Lu 2011 | 150 | 9 | 8 | 124 |
| 2011 | Said, H | Said 2011 | 117 | 9 | 0 | 99 |
| 2011 | Toihir, A | Toihir 2011 | 124 | 0 | 0 | 47 |
| 2010 | Muyoyeta, M | Muyoyeta 2010 | 224 | 2 | 1 | 396 |
| 2010 | Chihota, V | Chihota 2010 | 199 | 3 | 1 | 137 |
| 2009 | Ismail, N | Ismail 2009 | 71 | 0 | 2 | 23 |
| 2009 | Ngamlert K | Ngamlert 2009 | 225 | 1 | 7 | 14 |
| 2009 | Shen, G | Shen 2009 | 155 | 1 | 5 | 72 |
| 2007 | Purohit, M | Purohit 2007 | 141 | 1 | 12 | 49 |
| 2007 | Wang, J | Wang 2007 | 144 | 2 | 2 | 94 |
| 2006 | Mustafa, T | Mustafa 2006 | 32 | 3 | 3 | 17 |
| 2005 | Hillemann, D | Hillemann 2005 | 99 | 0 | 8 | 65 |
| 2004 | Hirano, K | Hirano 2004 | 381 | 0 | 3 | 161 |
| 2002 | Hasegawa, N. | Hasegawa 2002 | 158 | 6 | 0 | 140 |
| 1999 | Abe, C | Abe 1999 | 50 | 1 | 0 | 57 |
